# Hepatocyte Growth Factor Increases Osteopontin Expression in Human Osteoblasts through PI3K, Akt, c-Src, and AP-1 Signaling Pathway

**DOI:** 10.1371/journal.pone.0038378

**Published:** 2012-06-04

**Authors:** Hsien-Te Chen, Hsi-Kai Tsou, Chia-Hao Chang, Chih-Hsin Tang

**Affiliations:** 1 School of Chinese Medicine, College of Chinese Medicine, China Medical University, Taichung, Taiwan; 2 Department of Orthopedic Surgery, China Medical University Hospital, Taichung, Taiwan; 3 Department of Materials Science and Engineering, Feng Chia University, Taichung, Taiwan; 4 Department of Neurosurgery, Taichung Veterans General Hospital, Taichung, Taiwan; 5 Center for General Education, Jen-Teh Junior College of Medicine, Nursing and Management, Miaoli County, Taiwan; 6 Department of Orthopedic Surgery, Chang-Hwa Hospital, Department of Health Executive Yuan, Chang-Hwa County, Taiwan; 7 Department of Pharmacology, School of Medicine, China Medical University, Taichung, Taiwan; 8 Graduate Institute of Basic Medical Science, China Medical University, Taichung, Taiwan; The Hebrew University, Israel

## Abstract

**Background:**

Hepatocyte growth factor (HGF) has been demonstrated to stimulate osteoblast proliferation and participated bone remodeling. Osteopontin (OPN) is a secreted phosphoglycoprotein that belongs to the SIBLING family and is present during bone mineralization. However, the effects of HGF on OPN expression in human osteoblasts are large unknown.

**Methodology/Principal Findings:**

Here we found that HGF induced OPN expression in human osteoblasts dose-dependently. HGF-mediated OPN production was attenuated by c-Met inhibitor and siRNA. Pretreatment of osteoblasts with PI3K inhibitor (Ly294002), Akt inhibitor, c-Src inhibitor (PP2), or AP-1 inhibitor (curcumin) blocked the potentiating action of HGF. Stimulation of osteoblasts with HGF enhanced PI3K, Akt, and c-Src activation. In addition, incubation of cells with HGF also increased c-Jun phosphorylation, AP-1-luciferase activity, and c-Jun binding to the AP-1 element on the OPN promoter. HGF-mediated AP-1-luciferase activity and c-Jun binding to the AP-1 element was reduced by c-Met inhibitor, Ly294002, Akt inhibitor, and PP2.

**Conclusions/Significance:**

Our results suggest that the interaction between HGF and c-Met increases OPN expression in human osteoblasts via the PI3K, Akt, c-Src, c-Jun, and AP-1 signaling pathway.

## Introduction

Bone is a complex tissue composed of several cell types which are continuously undergoing a process of renewal and repair termed “bone remodeling”. The two major cell types responsible for bone remodeling are osteoclasts, which resorb bone, and osteoblasts, which form new bone. Bone remodeling is regulated by several systemic hormones (e.g., parathyroid hormone, 1, 25-dihydroxybitamin D_3_, sex hormones, and calcitonin), and local factors (e.g., nitric oxide, prostaglandins, growth factors, and cytokines) [1]. When resorption and formation of bone are not coordinated and bone breakdown overrides bone building, osteoporosis results [2]. Since new bone formation is primarily a function of the osteoblast, agents that act by either increasing the proliferation of cells of the osteoblastic lineage or inducing differentiation of the osteoblasts can enhance bone formation [3], [4].

Multiple anabolic signaling pathways are positively involved in controlling bone formation, such as bone morphogenetic proteins and osteopontin (OPN). OPN is a secreted phosphoglycoprotein that belongs to the SIBLING family and is present in various mineralized and soft tissues as well as in body fluids. OPN, a major member of the noncollagenous extracellular matrix secreted by osteoblasts and a cytokine involved in proliferation, apoptosis, and inflammatory signaling, has been implicated in bone remodeling [5]. It contains an RGDS motif, exhibits high affinity to calcium, and is produced by osteoblasts and osteoclasts. Despite the absence of a clear phenotype in OPN knockout mice, recent studies have implicated OPN in diverse biological processes, including development, wound healing, immunological responses, bone resorption, and calcification [6]. In addition, OPN-deficient mice also show reduced osteoblastic bone formation [7]. However, which mechanisms are regulated OPN expression in osteoblasts needs to be well elucidated.

Hepatocyte growth factor (HGF) was identified in the early 1980s [8], [9] and was subsequently determined to be a heterodimeric molecule composed of an alpha and beta chain [10]. The importance of HGF in organ development is demonstrated by HGF null mutation mice, which exhibit embryonic lethality [11]. HGF exhibits strong angiogenic properties through its ability to induce expression of vascular endothelial growth factor, another angiogenic factor, but also has angiogenic properties of its own [12]. Osteoblasts and osteoclasts express c-Met, the receptor for HGF and produce HGF [13]. HGF has been demonstrated to stimulate both osteoblast proliferation and osteoclast chemotactic migration [13]. In combination with vitamin D, HGF promotes osteoblast differentiation of vertebral bone marrow cells [14]. We hypothesized that HGF controls OPN expression in osteoblasts. This study was designed to test this hypothesis and also determine the precise signaling pathway.

**Figure 1:**
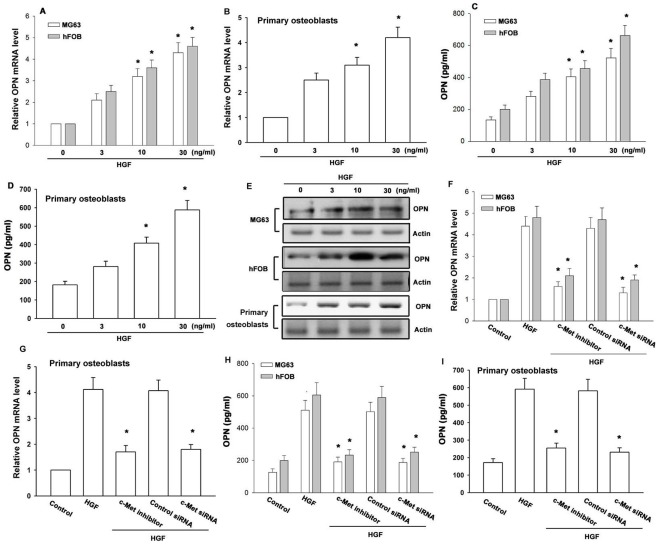
HGF increases OPN expression through c-Met receptor. (A&B) Cells were incubated with HGF for 24 h, and OPN mRNA was examined by qPCR (n = 4). (C-E) Osteoblasts were incubated with HGF for 24 h, and OPN protein was examined by ELISA and Western blotting (n = 4). (F-I) Cells were pretreated for 30 min with c-Met inhibitor or transfected with c-Met siRNA for 24 h followed by stimulation with HGF for 24 h, and OPN expression was examined by qPCR and ELISA (n = 4). *: p*<*0.05 as compared with basal level (A-D) or HGF-treated group (F-I).

Previous studies have shown that HGF modulates osteoblastic bone formation [13], [14]. HGF-mediated bone formation may involve activation of c-Met receptor. However, the effect of HGF on OPN (an osteoblastic formation gene) expression in human osteoblasts is mostly unknown. In this study, we found that HGF induces OPN expression in human osteoblasts. In addition, c-Met receptor, PI3K, Akt, c-Src, and AP-1 signaling pathways may be involved in the increase of OPN expression by HGF.

## Materials and Methods

### Materials

Anti-mouse and anti-rabbit IgG-conjugated horseradish peroxidase, rabbit polyclonal antibodies specific for β-actin, p-p85, p85, p-Akt, Akt, c-Src, p-c-Jun, c-Jun, and the small interfering RNAs (siRNAs) against c-Met, p85, Akt, c-Src, c-Jun, and a control for experiments using targeted siRNA transfection (each consists of a scrambled sequence that does not lead to specific degradation of any known cellular mRNA) were purchased from Santa Cruz Biotechnology (Santa Cruz, CA). Rabbit polyclonal antibodies specific for c-Src phosphorylated at Tyr^416^ was purchased from Cell Signaling and Neuroscience (Danvers, MA). Mouse monoclonal antibodies specific for OPN, c-Met, and OPN enzyme immunoassay kit were purchased from R&D Systems (Minneapolis, MN, USA). Recombinant human HGF was purchased from PeproTech (Rocky Hill, NJ). The AP-1 luciferase plasmid was purchased from Stratagene (La Jolla, CA). The p85 (Δp85; deletion of 35 amino acids from residues 479 to 513 of p85) and Akt (Akt K179A) dominant-negative mutants were gifts from Dr. W.M. Fu (National Taiwan University, Taipei, Taiwan). The c-Src dominant negative mutant was a gift from Dr. S. Parsons (University of Virginia Health System, Charlottesville, VA). The pSV-β-galactosidase vector and luciferase assay kit were purchased from Promega (Madison, WI). All other chemicals were purchased from Sigma-Aldrich (St. Louis, MO).

**Figure 2:**
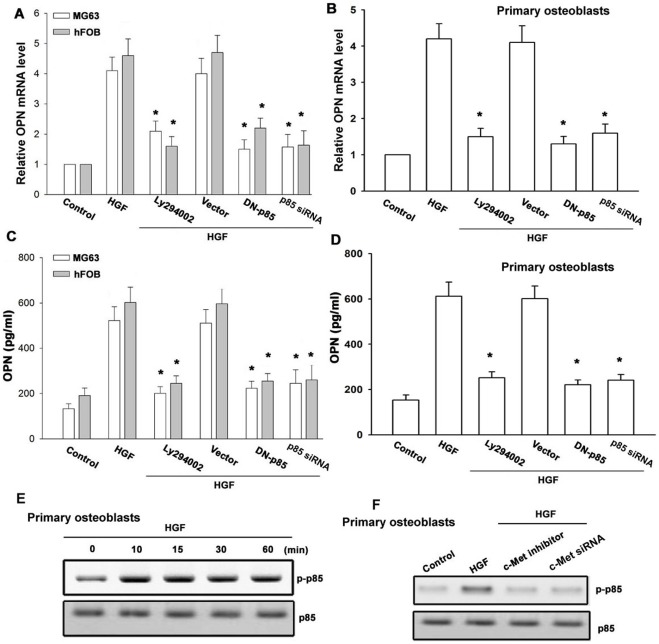
PI3K is involved in HGF-induced OPN expression. Cells were pretreated for 30 min with Ly294002 or transfected with p85 mutant and siRNA followed by stimulation with HGF for 24 h. Media and total RNA were collected, and the expression of OPN was analyzed with qPCR and ELISA (n = 4) (A-D). Primary osteoblasts were incubated with HGF for indicated time intervals, and p85 phosphorylation was examined by Western blotting (E). Primary osteoblasts were pretreated with c-Met inhibitor for 30 min or transfected with c-Met siRNA for 24 h followed by stimulation with HGF for 30 min, and p85 phosphorylation was determined by Western blotting (n = 5) (F). *: p*<*0.05 as compared with HGF-treated group.

**Figure 3:**
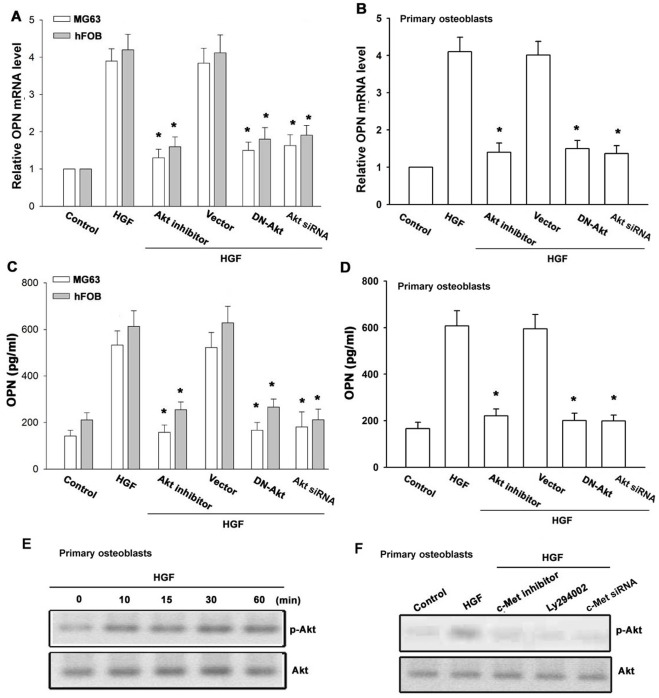
Akt is involved HGF-induced OPN expression. Cells were pretreated for 30 min with Akt inhibitor or transfected with Akt mutant and siRNA followed by stimulation with HGF for 24 h. Media and total RNA were collected, and the expression of OPN was analyzed with qPCR and ELISA (n = 4) (A–D). Primary osteoblasts were incubated with HGF for indicated time intervals, and Akt phosphorylation was examined by Western blotting (E). Primary osteoblasts cells were pretreated with c-Met inhibitor and Ly294002 for 30 min or transfected with c-Met siRNA for 24 h followed by stimulation with HGF for 30 min, and Akt phosphorylation was determined by Western blotting (F). *: p*<*0.05 as compared with HGF-treated group.

### Cell Culture

The MG-63 and hFOB cell lines were purchased from American Type Culture Collection (ATCC; Rockville, MD). The human osteoblast-like cell line MG-63 was cultured in MEM supplemented with 10% FBS and antibiotics (100 U/ml of penicillin G and 100 µg/ml of streptomycin). The conditionally immortalized human fetal osteoblastic cell line, hFOB, was maintained in a 1∶1 mixture of phenol-free DMEM/Ham’s F12 medium (GIBCO-BRL; Gaithersburg, MD) containing 10% FBS supplemented with geneticin (300 µg/ml) and antibiotics at 33.5°C, the permissive temperature for the expression of the large T antigen. All experiments with hFOB cells were carried out at the permissive temperature of 33.5°C.

**Figure 4:**
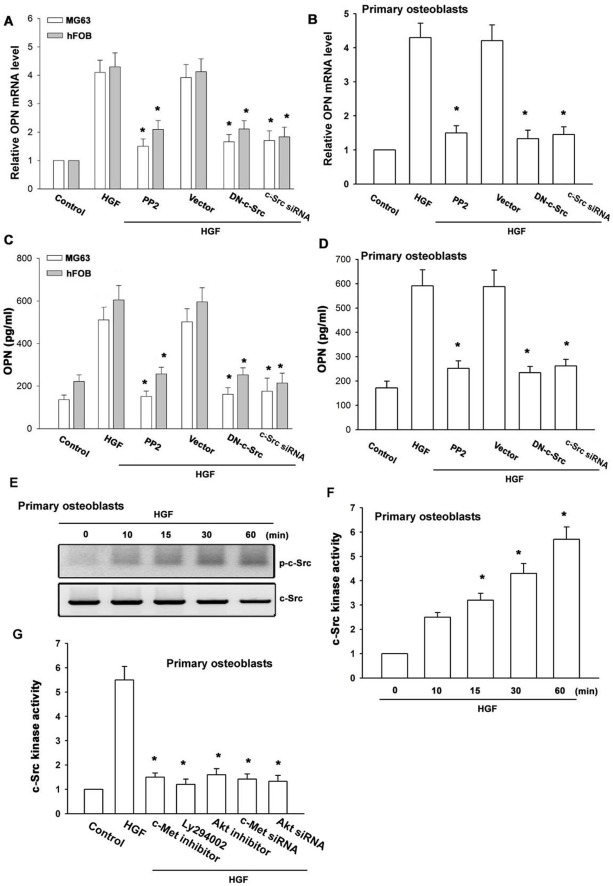
c-Src is involved in HGF-mediated OPN production in osteoblasts. Cells were pretreated for 30 min with PP2 or transfected with c-Src mutant and siRNA followed by stimulation with HGF for 24 h. Media and total RNA were collected, and the expression of OPN was analyzed with qPCR and ELISA (n = 5) (A–D). Primary osteoblasts were incubated with HGF for indicated time intervals, and c-Src phosphorylation was examined by Western blotting (E). Primary osteoblasts were incubated with HGF for indicated time intervals, and c-Src kinase activity was examined by c-Src kinase assay kit (F). Primary osteoblasts were pretreated with c-Met inhibitor, Ly294002, and Akt inhibitor for 30 min or transfected with c-Met and Akt siRNA for 24 h followed by stimulation with HGF for 30 min, and c-Src kinase activity was examined by c-Src kinase assay kit (G). *: p*<*0.05 as compared with basal level (F) or HGF-treated group (A-D&G).

Primary human osteoblasts were isolated from bone chips of ten 40–60-year-old donors who were generally healthy with no other bone disorders than hip dysplasia for which they received hip arthroplasty at China Medical University Hospital. The protocol for this study was approved by the Institutional Review Board at China Medical University Hospital and the informed consent was obtained from each donor. The osteoblasts were cultured in DMEM containing 100 µg/ml of ascorbic acid, non-essential amino acids, penicillin/streptomycin and 10% FBS. Cultures were maintained in a humidified atmosphere of 5% CO_2_ at 37°C.

**Figure 5:**
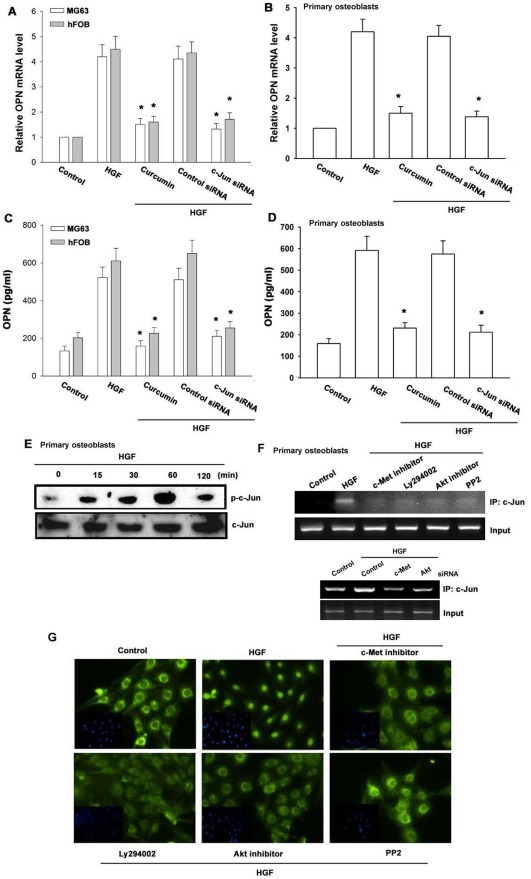
AP-1 is involved in the potentiation of OPN production by HGF. Cells were pretreated for 30 min with curcumin or transfected with c-Jun siRNA followed by stimulation with HGF for 24 h. Media and total RNA were collected, and the expression of OPN was analyzed with qPCR and ELISA (n = 5) (A–D). Primary osteoblasts were incubated with HGF for indicated time intervals, and c-Jun phosphorylation was determined by Western blotting (E). Primary osteoblasts were pretreated with c-Met inhibitor, Ly294002, Akt inhibitor, and PP2 or transfected with c-Met and Akt siRNA, and then stimulated with HGF for 120 min. A chromatin immunoprecipitation assay was then performed. The chromatin was immunoprecipitated with anti-c-Jun. One percent of the precipitated chromatin was assayed to verify equal loading (input) (F). MG63 cells were pretreated with c-Met inhibitor, Ly294002, Akt inhibitor, or PP2, and then stimulated with HGF for 120 min, and c-Jun immunofluorescence staining was examined (G). *: p*<*0.05 as compared with HGF-treated group.

### Measurement of OPN Production

Human osteoblasts were cultured in 24-well culture plates. After reaching confluency, cells were treated with HGF and then incubated in a humidified incubator at 37°C for 24 h. To examine the downstream signaling pathways involved in HGF treatment, cells were pretreated with various inhibitors for 30 min before addition of HGF (30 ng/ml) administration. After incubation, the medium was removed and stored at −80°C until the assay was performed. OPN in the medium was assayed using OPN enzyme immunoassay kits, according to the procedure described by the manufacturer.

**Figure 6:**
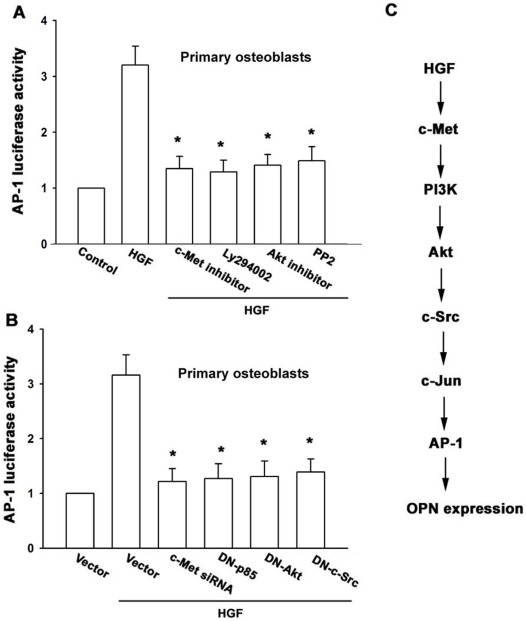
The c-Met, PI3K, Akt, and c-Src pathway is involved in HGF-induced AP-1 activation. (A&B) Primary osteoblasts were transfected with the AP-1-luciferase expression vector and then pretreated with c-Met inhibitor, Ly294002, Akt inhibitor, and PP2 or cotransfected with c-Met and c-Jun siRNA or p85, Akt and c-Src mutant before incubation with HGF for 24 h. Luciferase activity was then assayed (n = 4). *: p*<*0.05 as compared with HGF-treated group. (C) Schematic diagram of the signaling pathways involved in HGF-induced OPN expression in osteoblasts. HGF increases OPN expression by binding to the c-Met receptor and activating PI3K, Akt, and c-Src, which enhances binding of c-Jun to the AP-1 site. This results in the transactivation of OPN expression.

### Quantitative Real Time PCR

Total RNA was extracted from osteoblasts by using a TRIzol kit (MDBio Inc., Taipei, Taiwan). Two µg of total RNA was reverse transcribed into cDNA by using oligo(dT) primer [15], [16]. The quantitative real time PCR (qPCR) analysis was carried out using Taqman® one-step PCR Master Mix (Applied Biosystems, CA, USA). Two µl of total cDNA were added per 25-µl reaction with sequence-specific primers and Taqman® probes. Sequences for all target gene primers and probes were purchased commercially (GAPDH was used as internal control) (Applied Biosystems, Foster City, CA, USA). qPCR assays were carried out in triplicate on an StepOnePlus sequence detection system. The cycling conditions were 10-min polymerase activation at 95°C followed by 40 cycles at 95°C for 15 s and 60°C for 60 s. The threshold was set above the non-template control background and within the linear phase of target gene amplification to calculate the cycle number at which the transcript was detected (denoted C_T_).

### Western Blot Analysis

Cellular lysates were prepared as described [17], [18]. Proteins were resolved using SDS-PAGE and transferred to Immobilon polyvinyldifluoride membranes. The membranes were blocked with 4% BSA for 1 h at room temperature and then probed with rabbit antibodies against human p-p85, p85, p-Akt, Akt, p-c-Jun, or c-Jun (1∶1000) for 1 h at room temperature. After three washes, the blots were incubated with a donkey anti-rabbit peroxidase-conjugated secondary antibody (1∶1000) for 1 h at room temperature. The blots were visualized with enhanced chemiluminescence on Kodak X-OMAT LS film (Eastman Kodak, Rochester, NY).

### Kinase Activity Assay

c-Src activity was assessed by c-Src Kinase Activity Assay Kit (Abnova, Taipei, Taiwan) according to manufacturer’s instructions. Kinase activity kit is based on a solid-phase ELISA that uses a specific synthetic peptide as a substrate for c-Src and a polyclonal antibody that recognized the phosphorylated form of the substrate.

### Transfection and Reporter Gene Assay

Human osteoblasts were co-transfected with 0.8 µg AP-1 luciferase plasmid and 0.4 µg β-galactosidase expression vector. Cells were grown to 80% confluency in 12-well plates and then transfected on the following day with Lipofectamine 2000 (LF2000; Invitrogen). DNA and LF2000 were premixed for 20 min and then added to the cells. After 24 h of transfection, the cells were incubated with the indicated reagents. After a further 24 h of incubation, the medium was removed, and cells were washed once with cold PBS. To prepare lysates, 100 µl reporter lysis buffer (Promega, Madison, WI) was added to each well, and cells were scraped from dishes. The supernatant was collected after centrifugation at 13,000 rpm for 2 min. Aliquots of cell lysates (20 µl) containing equal amounts of protein (20–30 µg) were placed into wells of an opaque black 96-well microplate. An equal volume of luciferase substrate was added to all samples, and luminescence was measured in a microplate luminometer. The value of luciferase activity was normalized to the transfection efficiency, which was monitored by activity of the co-transfected β-galactosidase expression vector.

### Chromatin Immunoprecipitation Assay

Chromatin immunoprecipitation analysis was performed as described previously [19]. DNA immunoprecipitated with an anti-c-Jun Ab was purified and extracted with phenol-chloroform. The purified DNA pellet was subjected to PCR. PCR products were then resolved by 1.5% agarose gel electrophoresis and visualized with UV light. The primers 5′-TCT TCCTGG ATGCTGAATGC-3′ and 5′-CCA AGCCCT CCCAGA ATTTAA-3′ were utilized to amplify across the OPN promoter region [20].

### Statistics

The values given were means ± S.E.M and analyzed with one-way ANOVA. The difference was significant if the *p* value was <0.05.

## Results

### HGF Induces OPN Production in Human Osteoblasts

HGF has been reported to stimulate proliferation and differentiation of osteoblasts [13], [14]. To examine the effects of HGF on OPN expression, osteoblasts were exposed to HGF, and the mRNA levels of OPN was determined. Treatment of osteoblasts with HGF (3–30 ng/ml) for 24 h induced OPN mRNA levels (Fig. 1A&B). In addition, stimulation of osteoblasts with HGF also led to increased protein expression of OPN in a concentration-dependent manner by using ELISA and Western blotting (Fig. 1C–E). It has been reported that HGF exerts its effects through interaction with a specific receptor c-Met [21]. Pretreatment of osteoblasts with c-Met inhibitor reduced HGF-increased OPN expression (Fig. 1F–I). In addition, transfection of cells with c-Met siRNA also reduced HGF-increased OPN expression (Fig. 1F–I). Therefore, an interaction between HGF and c-Met is very important for OPN production in human osteoblasts.

### The Signaling Pathways of PI3K and Akt are Involved in the Potentiating Action of HGF

PI3K/Akt signaling pathway can be activated by a variety of growth factors, such as insulin and HGF [22], [23]. To determine whether PI3K was involved in HGF triggered OPN expression, osteoblasts were pretreated with Ly294002, a PI3K inhibitor for 30 min and then incubated with HGF for 24 h. As shown in Fig. 2A–D, pretreatment with Ly294002 reduced HGF-induced OPN expression. Transfection of cells with p85 mutant or siRNA also reduced HGF-induced OPN expression (Fig. 2A–D). We then directly measured p85 phosphorylation in response to HGF. Stimulation of primary osteoblasts led to a significant increase in phosphorylation of PI3K (Fig. 2E). In addition, treatment with c-Met inhibitor or transfection with c-Met siRNA reduced HGF-mediated p85 phosphorylation (Fig. 2F). Furthermore, pretreatment of cells with Akt inhibitor or transfection with Akt mutant and siRNA for 24 h markedly attenuated the HGF-induced OPN production (Fig. 3A–D). Akt phosphorylation in response to HGF was then measured. As shown in Fig. 3E, treatment of primary osteoblasts with HGF resulted in a time-dependent phosphorylation of Akt. Pretreatment of cells with c-Met inhibitor and Ly294002 or transfection with c-Met siRNA inhibited the HGF-induced Akt phosphorylation (Fig. 3F). Taken together, these results indicate that the PI3K and Akt pathways are involved in HGF-induced OPN production.

### c-Src is Involved in the HGF-mediated OPN Expression in Osteoblasts

PI3K/Akt-dependent c-Src activation is involved in the regulation of gene expression [24]. Therefore, we investigated the role of Src in mediating HGF-induced OPN expression with the specific Src inhibitor PP2. As shown in Figure 4A–D, HGF-induced OPN expression was markedly attenuated by pretreatment of cells for 30 min with PP2 or transfected of cells for 24 h with c-Src mutant and siRNA. The major phosphorylation site of c-Src at the Tyr^416^ residue results in activation from c-Src autophosphorylation [25]. To directly confirm the crucial role of c-Src in OPN expression, we measured the level of c-Src phosphorylation at Tyr^416^ in response to HGF. As shown in Figure 4E, treatment of primary osteoblasts with HGF resulted in a time-dependent phosphorylation of c-Src at Tyr^416^. In addition, HGF also increased c-Src kinase activity (Fig. 4F). Pretreatment of primary osteoblasts with c-Met inhibitor, Ly294002, and Akt inhibitor or transfection with c-Met and Akt siRNA markedly inhibited the HGF-induced c-Src kinase activity (Fig. 4G). Based on these results, HGF appears to act through a signaling pathway involving c-Met receptor, PI3K, Akt, and c-Src to enhance OPN expression in human osteoblasts.

### Involvement of AP-1 in HGF-induced OPN Expression

The promoter region of human OPN contains AP-1 binding site [20]. To examine the role of the AP-1 binding site in HGF-mediated OPN expression, the AP-1 inhibitor curcumin was used. Pretreatment of cells with curcumin reduced HGF-enhanced OPN expression (Fig. 5A–D). AP-1 activation was further evaluated by analyzing the phosphorylation of c-Jun as well as by a chromatin immunoprecipitation assay. Treatment of primary osteoblasts with HGF increased c-Jun phosphorylation (Fig. 5E). In addition, transfection of cells with c-Jun siRNA suppressed HGF-induced OPN expression (Fig. 5A–D). We next investigated whether c-Jun binds to the AP-1 element on the OPN promoter after HGF stimulation. The *in vivo* recruitment of c-Jun to the OPN promoter was assessed via chromatin immunoprecipitation assay. *In vivo* binding of c-Jun to the AP-1 element of the OPN promoter occurred after HGF stimulation (Fig. 5F). The binding of c-Jun to the AP-1 element by HGF was attenuated by c-Met inhibitor, Ly294002, Akt inhibitor, and PP2 or c-Met and Akt siRNA (Fig. 5F). In addition, pretreatment of cells with c-Met inhibitor, Ly294002, Akt inhibitor, and PP2 also reduced HGF-induced accumulation of c-Jun into the nucleus (Fig. 5G).

To further confirm that the AP-1 element is involved in HGF-induced OPN expression, we performed transient transfection with AP-1 promoter-luciferase constructs. Primary osteoblasts incubated with HGF showed a 3.2-fold increase in AP-1 promoter activity. The increase in AP-1 activity by HGF was antagonized by c-Met inhibitor, Ly294002, Akt inhibitor, and PP2 or c-Met and c-Jun siRNA or p85, Akt, and c-Src mutant (Fig. 6A&B). Taken together, these data suggest that the activation of the c-Met, PI3K, Akt, c-Src, c-Jun, and AP-1 pathway is required for the HGF-induced increase in OPN expression in human osteoblasts.

## Discussion

In this study with osteoblasts, elevation of OPN mRNA and protein levels followed recombinant HGF protein treatment through c-Met receptor induction of the PI3K, Akt, and c-Src signaling pathway. Our findings provide the first evidence that HGF increased OPN expression, providing a link and molecular mechanism between HGF family and OPN in the physiology of bone. In this study, we provided the data from primary osteoblasts to examine the molecular mechanism between HGF and OPN. However, the interactions of these two molecules in the *in vivo* scenario are large unknown. In next steps, we try to establish the HGF knockout mice and to examine the OPN expression in the bone. These data will provide the *in vivo* data of present study.

HGF plays an essential role in the development and regeneration of the liver and also stimulates the growth, motility, and morphogenesis of a variety of cell types [26]. Although initially thought to be of mesodermal origin, HGF is expressed almost ubiquitously, including by osteoblasts [27]. It has been postulated to participate in bone remodeling and formation [13]. Previously reported that HGF (10 ng/ml) combined with vitamin D_3_ may have an autocrine and/or paracrine effect on the osteogenic maturation, as well as on bone repair and remodeling [14]. In this study, we found that high concentration of HGF (30 ng/ml) induced osteogenic gene (OPN) expression. These data imply that using high concentration HGF may increases osteogenic maturation without vitamin D_3_ application. However, this hypothesis is needs further examination. The biological activity of HGF is mediated by binding to cell surface c-Met receptor. Here, we confirmed that c-Met receptor is required for HGF-induced OPN expression. Pretreatment of cells with c-Met inhibitor reduced HGF-induced OPN expression. This was further confirmed by the result that the c-Met siRNA inhibited the enhancement of OPN production by HGF. Therefore, the interaction between HGF and c-Met is very important for OPN production by human osteoblasts.

PI3K/Akt have been characterized at the molecular level and have been found to mediate several cellular molecular responses [28]. Phosphorylation of the p85 subunit is required for activation of the p110 catalytic subunit of PI3K [29]. We found HGF-enhanced the p85 subunit phosphorylation in human osteoblasts. Pretreatment of cells with PI3K inhibitor Ly294002 antagonized an increase in OPN expression by HGF stimulation. This was further confirmed by the result that the dominant-negative mutant and siRNA of p85 inhibited the enhancement of OPN by HGF. Moreover, we also found that HGF activated Akt Ser^473^ phosphorylation, while Akt inhibitor and Akt mutant or siRNA inhibited HGF-mediated OPN expression. Our data indicates that PI3K/Akt could play an important role in the expression of OPN in human osteoblasts.

Src, a tyrosin kinase, plays a critical role in the induction of gene transcription [30]. Because c-Src is a downstream effector of PI3K/Akt [24], we examined the potential role of c-Src in the signaling pathway HGF-induced OPN expression. Treatment of cells with c-Src inhibitor PP2 or transfection of cells with c-Src mutant and siRNA reduced HGF-mediated OPN expression. In addition, we also found that treatment of osteoblasts with HGF induced increases in c-Src phosphorylation and kinase activity. These effects were inhibited by c-Met inhibitor, Ly294002, and Akt inhibitor, indicating the involvement of PI3K/Akt-dependent c-Src activation in HGF-mediated OPN induction.

There are several binding sites for a number of transcription factors including NF-κB, Sp-1, and AP-1 in the 5′ region of the OPN gene [20]. Recent studies of the OPN promoter have demonstrated that OPN induction by several transcription factors occurs in a highly stimulus-specific or cell-specific manner [31], [32]. The results of our current study show that AP-1 activation contributes to HGF-induced OPN expression in osteoblasts. Pretreatment of cells with an AP-1 inhibitor curcumin reduced HGF-increased OPN expression. Therefore, the AP-1 binding site is likely to be the most important site for HGF-induced OPN production. The AP-1 sequence binds to members of the Jun and Fos families of transcription factors. These nuclear proteins interact with the AP-1 site as Jun homodimers or Jun-Fos heterodimers formed by protein dimerization through their leucine zipper motifs. The results of our study show that HGF induced c-Jun phosphorylation. In addition, c-Jun siRNA abolished HGF-induced OPN expression in osteoblasts. Therefore, c-Jun activation mediates by HGF-increased OPN expression. Furthermore, HGF increased the binding of c-Jun to the AP-1 element within the OPN promoter, as shown by a chromatin immunoprecipitation assay. Binding of c-Jun to the AP-1 element was attenuated by c-Met inhibitor, Ly294002, Akt inhibitor, and PP2. Using transient transfection with AP-1-luciferase as an indicator of AP-1 activity, we also found that HGF induced an increase in AP-1 activity. In addition, c-Met inhibitor, Ly294002, Akt inhibitor, and PP2 or c-Met and c-Jun siRNA or p85, Akt, and c-Src mutant reduced HGF-increased AP-1 promoter activity. These results indicate that HGF may act through the c-Met receptor, PI3K, Akt, c-Src, c-Jun, and AP-1 pathway to induce OPN production in human osteoblasts.

In conclusion, we explored the signaling pathway involved in HGF-induced OPN expression in human osteoblasts. We found that HGF increased OPN expression by binding to the c-Met receptor and activating PI3K, Akt and c-Src, which enhanced binding of c-Jun to the AP-1 site and resulted in the transactivation of OPN expression (Fig. 6C).
